# Five traditional Nigerian Polyherbal remedies protect against high fructose fed, Streptozotocin-induced type 2 diabetes in male Wistar rats

**DOI:** 10.1186/s12906-018-2225-6

**Published:** 2018-05-16

**Authors:** O. E. Kale, O. B. Akinpelu, A. A. Bakare, F. O. Yusuf, R. Gomba, D. C. Araka, T. O. Ogundare, A. C. Okolie, O. Adebawo, O. Odutola

**Affiliations:** 1 Department of Pharmacology, Benjamin S. Carson (Snr.) School of Medicine, Babcock University, Ilishan-Remo, Ogun State, PMB, Ikeja, 21244 Nigeria; 2 Department of Biochemistry, Benjamin S. Carson (Snr.) School of Medicine, Babcock University, Ilishan-Remo, Ogun State, PMB, Ikeja, 21244 Nigeria

**Keywords:** Type 2 diabetes mellitus, Polyherbal, Fructose, Streptozotocin, Oxidative stress

## Abstract

**Background:**

This present study sought to assess the modulatory effects of five Nigerian traditional polyherbal in high fructose-fed, streptozotocin-induced (HF-STZ) Type 2 diabetes (T2D) in rats. T2D was achieved via fructose feeding (20%^*W*/*V*^) ad libitum for 2 weeks and streptozotocin (STZ, 40 mg/kg) (15th Day) intraperitoneally.

**Methods:**

Seventy-two hours after STZ injection, fourty-eight diabetic rats were divided into eight of 6 rats/group: Diabetic normal untreated, glibenclamide (GBLI, 0.07 mL/kg) or yoyo (YB, 0.43), ruzu (RB, 0.08), fajik (FJB, 0.20), oroki (OB, 0.16), and fidson (FB, 0.43)/ mL/kg bitters respectively. Controls normal and diabetic untreated groups received intragastric carboxylmethylcellulose (CMC, 1 mL/kg) for eleven days.

**Results:**

T2D was characterized in rats by an increased (*p* < 0.001–0.05) blood glucose levels (BGL), total cholesterol, triglycerides, low-density lipoprotein and alanine aminotransferase compared with control CMC group. Similarly, hepatic and pancreatic malondialdehyde (MDA) were increased by 180 and 97% respectively. Polyherbal treatments demonstrated efficacies on BGL as follow: YB (55.6%, 160.7 mg/dL); RB (59.7%, 145.2 mg/dL); FJB (59.8%, 243.4 mg/dL); OB (60.8%, 194.5 mg/dL) and FB (61.3%, 203.3 mg/dL) respectively by day 11 (versus GBLI, 65.1%) compared with control untreated diabetic rats. Also, elevated TC, LDL cholesterol, ALT were lowered (*p* < 0.05) by YB, FJB, and FB respectively in rats. YB, FJB, and OB lowered MDA levels in treated rats. Further, YB, RB, FJB and FB restored changes in liver, and pancreas histopathology. Predominant non-polar bioactive include oleic, hexadecanoic, octadecanoic among others following gas chromatography-mass spectrophotometry analyses.

**Conclusion:**

Overall, these present results demonstrate anti-hyperglycemic potentials, although with cautions, of some polyherbal in T2D rats, which may, in part, be antioxidants mediated.

## Background

In most developing countries and even some developed, over the counter use of polyherbal is on the high side and the manufacturers claimed a complete cure for diabetes mellitus (type 2 diabetes, T2D). A polyherbal mixture is composed of different plants constituents and unpurified extracts with medicinal properties in maintaining good health and for treatment of different aliments [1]. As a result of cost effectiveness and the notions for fewer side effects, these concoctions have been reportedly used in the treatment of metabolic syndrome risk factors particularly T2D, although, not many have undergone careful scientific evaluation [2]. T2D is an endocrine pathological disorder characterized by two significant conditions resulting from defects in insulin secretion or reduced sensitivity of the tissue to insulin (insulin resistance) and pancreas β-cells dysfunction [3]. Other metabolic syndrome comorbidities have also been found in the vicinity of T2D and may be responsible for increased metabolic syndrome risks factors [1, 2]. Several medicinal plants have birthed active drugs which are considered as useful resources capable of preventing and improving metabolic syndrome diseases [3]. Up to now, there have been increases in the search for new anti-diabetic agents which are cheaper with greater effectiveness and lesser side effects due to the fact that many of the available synthetic oral hypoglycemic agents are costly and produce predictable adverse drug reactions [4]. Also, some noxious actions of hypoglycemic agents have led many to turn to the use of herbs in the treatment of diabetes [5]. Yearly, several herbs are scientifically reported to have anti-diabetic effects as well as significant antioxidant activity and this has led to the formulation of polyherbal based on these hypotheses. In respect, a combination of herbs is believed to work synergistically and may have a more beneficial effect than in single preparation [6]. The five most commonly paraded bitters in Nigeria for T2D are Yoyo bitters (YB), Oroki herbal mixture (OB), Ruzu Bitters (RB), Fijk flusher (FB), and Fidson Bitter (FB) respectively. Although, each of this preparation has claimed for several indications, however, this study sought to verify this aspect predicated solely on T2D. Interestingly, for any medicinal plant to be present and used in a formulation, many of the individual constituents of these agents have been investigated extensively and reported in the literature. The following compounds have been reported given these combinations with very wide applications: YB (*Acinos arvensis*, *Chenopodium murale, Citrus aurantifolia, Aloe vera* and *Cinnamomum aromaticum* [7], OB (*Sorghum bicolor*, *Khaya grandifoliola*, *Cassia sieberiana*, *Staudtia stipitata*, *Alstonia cognensis*, *Ocimum basillicum*, *Mangifera indica*, *Cythula prostrate*, *Securidaca longepedunculata*, *Saccharum officinarum* and water [8]. RB (*Uvarie chamae, Curculigo pilosa* and *Colocythis citrullis*) [9], FJB (*Cassia alata, Citrus medica var. acida* (Roxb.)*, Aloe barbaris, Aloe vera, Cassia angustifolia*) and FB (*Ginseng, Phyllanthus niruri, Aloe vera, Tephrosia purpurea, Eclipta alba, Swertia chirata* (Buch-Ham.)*, Casssia angustifolia, Cinnamomum zeylanicum*). In respect, some of their characteristic phytomedicines or bioactive components have also been confirmed by different studies. For instance, *Acinos arvensis* **(**Lamiaceae**)** commonly referred to as basil thyme has been reported to contain a number of compounds such as germacrene, hexadecanoic acid, β-bourbonene, pulegone, izomenthone, phytol, linarin [10]. *Aloe vera* (Liliaceae) is one of the oldest medicinal plants with diversifying medicinal effects which have been explored and reviewed [11]. Many of its compounds possess anti-tumor, anti-arthritic, anti-rheumatoid, anti-cancer, anti-diabetic, antioxidant, anti-microbial, anti-viral, anti-hyperlipidermic, anti-ulcer, hepatoprotective and immunomodulatory properties [12]. Glucomannan, a water-soluble fiber from this plant, possesses anti-hyperglycemic and insulin sensitizing activity [13]. Thus, evidence abounds that *Aloe vera* improved the survival of islet cells of rat pancreas, increased insulin levels and decreased the production of reactive oxygen species [12, 14]. *Citrus aurantifolia* (Rutaceae) is referred to as key lime or “osan wewe” in South Western Nigeria [15]. Lime juice has numerous numbers of nutrients and phytochemical substances such as citric acid, ascorbic acid, minerals, and flavonoids. Further derivative includes hesperedin, apigenin, naringenin, quercetin, rutin etc. [16]. It has been reported to possess antibacterial, anti-cancer, anti-diabetic, anti-fungal, anti-hypertensive, anti-inflammatory, anti-lipidemic and antioxidant properties [16]. *Chenopodium murale* (Chenopodiaceae) grows on waste a land which is commonly referred to as nettle- leaf goose foot. *Chenopodium murale* has yielded analgesics, anti-inflammatory, anti-fungal, anti-bacterial, anti-oxidant, hypotensive and hepatoprotective molecules [17]. *Cinnamomum aromaticum*, commonly called *Cinnamon cassia* or Chinese cinnamon belongs to the Lauraceae family and it serves as a spice, flavoring agent, preservative, oral health agent as well as an anti-termitic, nematicidal, insecticidal [18]. Phytochemical studies have revealed the presence of volatile oils such as cinnamaldehyde, weitechin, cinnamylacetate, cinnamyl alcohol, cinnamic acid, lignans. Cinnamaldehyde is effective against metabolic disorder and diabetes-induced renal damage. *Cinnamomum aromaticum* shows biological effects such as anti-microbial, antioxidant, anti-inflammatory, anti-ulcerogenic, anticancer, analgesic, lipid-lowering, cardiovascular disease lowering, coagulating and anti-diabetic effects [18]. It has also been reported to have activity against neurological disorders such as Parkinson’s and Alzheimer’s disease. *Curculigo pilosa* of the genus curculigo belongs to the family Hypoxidacea and also known as ground squirrel groundnut or “Epakun” in Yoruba land, Nigeria [19]. *Curculigo pilosa* is used in the treatment of gastrointestinal diseases and cardiovascular heart related diseases due to its good anti-oxidant properties [19]. *Colocynthis citrullus* belongs to the family Cucurbitaceae which is referred to locally as bitter apple cucumber and Egusi baara by the Yorubas. It has been reported to enhance the activity of glucokinase/ hexokinase pathway in the liver [20]. *Phyllanthus niruri* aids memory enhancement, help to reduce fatigue and possess anti-ageing and anti-stress properties [21]. Also, *Aloe vera*, *Tephrosia purpurea* and *Eclipta alba* have hepatoprotection, blood cleansing synergy and also help in managing gastrointestinal integrity and heart burn [22]. *Eclipta alba*, *Aloe vera*, *Tephrosia purpurea* and *Phyllanthus niruri* together offer hepatoprotection weight loss therapy. *Swertia chirata*, *Phyllanthus niruri*, *Casssia angustifolia*, *Cinnamomum zeylanicum*, *Aloe vera* and *Tephrosia purpurea* are said to aid digestion, regulate bowel movement and for correction of urinary disorders [23]. *Cinnamomum zeylanicum*, *Swerta chirata*, *Aloe vera* and ginseng combination will help regulate blood glucose levels [23]. Despite the great relevance placed on polyherbal, several of these popular plants used traditionally for the treatment of diabetes have received criticism elsewhere [24, 25]. Thus**,** standardization of polyherbal is essential for several reasons. These include verify the manufacturers’ claims, assess the quality of products as well as document the detail toxicological profiles based on the concentration of their active principles [26]. Therefore, this study assessed five Nigerian popular yoyo, ruzu, fajik, oroki, and fidson herbal remedies in high fructose-fed, streptozotocin-induced T2D in male Wistar rats.

## Methods

### Drugs and chemicals

Yoyo bitters® was purchased at Romitel Pharmacy limited at Mowe, Ogun State, Nigeria. Ruzu Bitters® was purchase from Ruzu Natural Health Products and services (Egan-Igando, Lagos Nigeria). Oroki herbal mixture® was purchased from Nured industrial and commercial company limited located in Lagos Nigeria. Fijk Flusher® was purchased from De-Fayus Organization Igando Market Igando Rd., Alimosho, Lagos (Nigeria). Fidson Bitters® was purchased from Fidson Pharmaceutical Limited, Nigeria. Streptozotocin was purchased from Sigma Aldrich respectively and Fructose from Burgoyne Reagents (India), Reduced glutathione (GSH), metaphosphoric acid and trichloroacetic acid (TCA) were purchased from J.I. Baker (Center Valley, PA, U.S.A.). Thiobarbituric acid (TBA) was purchased from Sigma Chemical Company (USA). Alanine aminotransferase (ALT), aspartate aminotransferase (AST), alkaline phosphatase (ALP), total cholesterol (TC), and triglyceride (TG) assay kits were obtained from Randox Laboratory (Crumlin, UK), 5^l^, 5^l^-dithiobis-2-nitrobenzoate (Ellman’s reagent) from Sigma (USA) and sodium hydroxide from Merck (Germany). Other chemicals and reagents used were of analytical grade.

### Experimental animals

The study was carried out in compliance with established guidelines for biomedical research as approved by the Babcock University, Ogun State, Nigeria in conjunction with the organization for Animal Care and Use in Research, Education and Testing (ACURET.ORG). An ethical clearance was obtained from the Babcock University Human Research Ethics Committee (BUHREC 308/17). The study was carried out in the Department of Biochemistry, Benjamin Carson (Snr.) School of Medicine, Babcock University, Nigeria. Healthy adult male Wistar rats (160 ± 40 g) were obtained from a commercial private colony in Ibadan, Oyo-State, Nigeria and housed in the Babcock University Laboratory animal (Ilishan, Ogun State, Nigeria) facility. They were housed in a unisexual group of 4 in metallic cages (60 × 45 × 25 cm) under a reversed light-dark cycle (12 h/ 12 dark scheduled) and controlled temperature (22 ± 3 °C). The animals were acclimatized for 2 weeks. They were fed with commercially available pelleted diet (Vita Feeds, Jos, Plateau State, Nigeria) and water ad libitum during the period of acclimatization and throughout the period of the experiment. The investigation conforms to the Guide for the Care and Use of Laboratory Animals published by the U. S. National Institutes of Health (NIH Publication No. 85–23, revised 1996) for studies involving experimental animals and the procedures as documented by Kilkenny et al. [27] for reporting animal research.

### Extraction and bioactive compound identification

The fractionation process was carried out according to the method described by Onyeaghala et al. [28] with slight modifications. Briefly, 2 × 200 mL of the bitters was exhaustively extracted using n-hexane. The extraction was carried out in a ratio of 1:1 (polyherbal: hexane). The vortexed mixture of bitters and hexane was put into a separating funnel, vortexes and allowed to stand for about 45 min to ensure complete extraction of the non-polar components. The non-polar portion was separated by funnels, concentrated at 40 °C under reduced pressure using a rotary evaporator [29] and stored at − 4 °C until needed. Gas Chromatography-Mass Spectrometry (GC-MS) analysis of polyherbal from non-polar components was carried out using an Agilent HP- 7890A gas chromatograph (Agilent Technologies, Palo Alto, CA, USA) with HP-5MS 5% phenylmethylsiloxane capillary column (30 m × 0.25 mm, 0.25 lm film thickness; Restek, Bellefonte, PA) equipped with an MSD detector and characterized as previously described (Adams, 2001) and Proestos et al. (2006) with some modifications as reported elsewhere Okolie et al. [30].

### Induction of diabetes

T2D was achieved via the method of Wilson & Islam [31] as modified by Okolie et al. [30] by fructose feeding (20%^*W*/*V*^) ad libitum for 2 weeks and streptozotocin (40 mg/kg *i.p.*) to rats. Seventy-two house following STZ injection, fourty-eight (48) diabetic rats were divided into eight of 6 rats/group: Diabetic normal untreated, glibenclamide (0.07 mg/kg), and yoyo (YB, 0.43), ruzu (RB, 0.08), fajik (FB, 0.20), oroki (OB, 0.16), and fidson (FB, 0.43), mL/kg bitters respectively. All bitters were administered via the oral route. Controls normal and diabetic untreated groups received intragastric carboxylmethylcellulose (1 mL/kg) for two weeks.

### Necropsy

Animals were sacrificed by cervical dislocation 24 h after the last treatment, and blood was collected by cardiac puncture into plain bottles. Serum was separated by centrifugation at 4200 rpm at room temperature for 5 min. The pancreas and liver were carefully excised, cleared of adhering tissues, and weighed. Weight was recorded in grams and expressed as g/g body weight. A small portion of the excised pancreas and liver were fixed in 10% formaldehyde and subsequently prepared for histology. The remaining portion of the excised pancreas and liver were weighed and homogenized in four volumes of 100 mM of phosphate buffer (pH 7.4). The plasma and liver homogenates obtained from each animal were then analyzed to assess pancreas and liver function and other biochemical parameters.

### Biochemical assessment of hepatic function, lipid parameters, and antioxidants enzymes

Serum aspartate and alanine aminotransferases (AST and ALT) and alkaline phosphatase (ALP) activities were assessed for liver function. AST and ALT activities were determined according to the principle described by Reitman and Frankel [32] while the ALP activity was carried out according to the method described by Roy [33]. Total Cholesterol (TC) and Triglyceride (TG) concentrations were estimated following the principle described by Trinder [34] using commercial kits obtained from Randox Laboratories Ltd. (Crumlin, UK). Uric acid was also determined using Randox kit following the principle described by Fossati et al. [35]. The method described by Warnick and Albers [36] was used to determine High-Density Lipoprotein (HDL) while Freidewald formula [37] was used to extrapolate serum low-density lipoprotein (LDL). GSH level was estimated at 412 nm following the method of Beutler et al. [38]. Lipid peroxidation was estimated spectrophotometrically by the thiobarbituric acid reactive substance (TBARS) method as described by Varshney and Kale [39] and expressed in terms of malondialdehyde (MDA) formed per mg protein.

### Statistic

All data were expressed as mean ± S.E.M. Significant differences among the group were determined by T-test and one-way analysis of variance (ANOVA) using statistical package for social science student (version 20). Data were converted to figures using Graph Pad Prism (6.0). Results were considered to be significant at *p* ≤ 0.05.

## Results

### The effects of polyherbal on fasting blood glucose (FBGL) levels

**Fig. 1:**
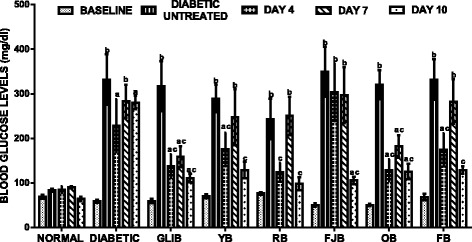
effects of polyherbals on fasting blood glucose (FBGL) levels in normal and diabetic rats. Results represented as Mean ± S.E.M. *n* = 6. ^*a*^*p* < 0.05 or ^b^ *<* 0.001 different from control normal CMC group. ^*c*^*p* < 0.05 or ^d^ *<* 0.001 different from control Untreated Diabetic group. CMC: Carboxylmethyl cellulose; YB: Yoyo bitters; RB: Ruzu bitters; FB: fajik bitters; OB: Oroki bitters; FB: Fidson bitters

Figure 1 shows effects of polyherbal on fasting blood glucose (FBGL) levels in normal and diabetic rats. The control normal CMC group, baseline, shows no significant change in FBGLs in all rats. However, at 72 h following STZ intoxication to high fructose fed rats, there were increased FBGL (*p* < 0.001–0.05) when compared with control CMC group. However, following treatments, glibenclamide (GBLI), an anti-diabetic drug, lowered FBGL (*p* < 0.05) by 56.5% (day 4), 50% (day 7) and 65.1% (day 11) respectively when compared with control untreated diabetic rats. Similarly, polyherbal demonstrated efficacies as follow: YB 39.4% (day 4), 14.5% (day 7), 55.6% (day 11); RB 49.2% (day 4), 2.8% (day 7), 59.7% (day 11); FJB 13.2% (day 4), 15.1% (day 7), 69.8% (day 11); OB 59.8% (day 4), 43.2% (day 7), 60.8% (day 11) and FB 47.5% (day 4), 14.9% (day 7), 61.3% (day 11) respectively compared with control untreated diabetic rats. FBGL remains unchanged in untreated diabetic rats when compared with baseline.

### The effects of polyherbal on body weight

**Fig. 2:**
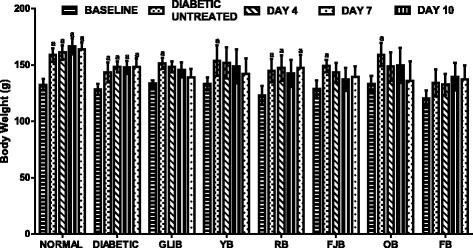
effects of polyherbals on body weight in normal and diabetic rats. Results represented as Mean ± S.E.M. n = 6. ^*a*^*p* < 0.05 different from control normal CMC group. CMC: Carboxylmethyl cellulose; YB: Yoyo bitters; RB: Ruzu bitters; FB: fajik bitters; OB: Oroki bitters; FB: Fidson bitters

Figure 2 shows effects of polyherbal on body weight in normal and diabetic rats. The baseline shows no significant change in body weight of rats. Assessment of body weight on day 4, 7 and 10th of treatments did not show any significant change in body weight when compared with controls. However, slight but insignificantly increased (*p* > 0.05) body weight in rats treated with OB (day 4, 19.4%), RB (day 7, 18.1%) and YB (day 10, 18.1%) when compared with control normal CMC group.

### The effects of polyherbal on lipid parameters

**Fig. 3:**
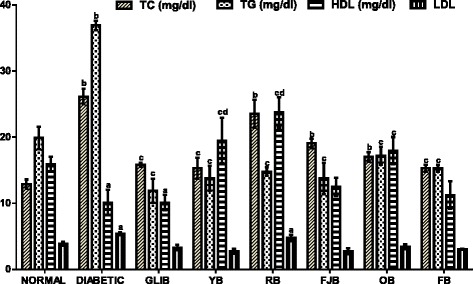
effects of polyherbals on lipid parameters in normal and diabetic rats. Results represented as Mean ± S.E.M. n = 6. ^*a*^*p* < 0.05 or ^b^ *<* 0.001 different from control normal CMC group. ^*c*^*p* < 0.05 or ^d^ *<* 0.001 different from control Untreated Diabetic group. CMC: Carboxylmethyl cellulose; YB: Yoyo bitters; RB: Ruzu bitters; FB: fajik bitters; OB: Oroki bitters; FB: Fidson bitters

Figure 3 results show effects of polyherbal on lipid parameters in normal and diabetic rats. Diabetic rats showed increased (*p* < 0.05) in serum total cholesterol (102.7%), triglycerides (85.4%), and low density lipoprotein (38.5%) when compared with control normal rats. Also, RB, FJB, OB elevated TC by 82.6, 47.8 and 41.6% respectively. In addition, RB increased (*p* > 0.05) LDL in treated rats. However, TG was reduced in treated rats: Glib (40.2%, *p* < 0.05), YB (30.2%, p < 0.05), RB (25.6%, p > 0.05), FJB (30.9%, p < 0.05), OB (17.2%, p > 0.05) and FB (23.1%, p > 0.05) respectively. YB, RB and OB increased HDL by 22.5% (*p* > 0.05), 49.3% (*p* < 0.05) and 12.8% (*p* > 0.05) respectively when compared with control normal rats. In addition, TC and LDL cholesterol levels were reduced (*p* < 0.05) by GLIB (28.1, 38.9%), YB (41.5, 48.9%), FJB (27.1, 49.1%), OB (34.99, 36.3%), and FB (41.5, 43.3%) by respectively when compared with control untreated diabetic group. YB, RB and OB increased (p < 0.05) HDL by 92.6, 134.7 and 77.4% respectively in the treated rats when compared with control untreated diabetic group.

### The effects of different polyherbal on liver function enzymes

**Fig. 4:**
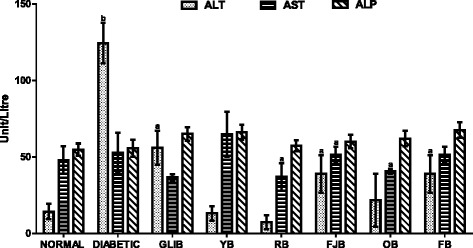
effects of polyherbals on plasma ALT, AST and ALP levels in normal and diabetic rats. Results represented as Mean ± S.E.M. n = 6. ^*a*^*p* < 0.05 or ^b^ *<* 0.001 different from control normal CMC group. ^*c*^*p* < 0.05 or ^d^ *<* 0.001 different from control Untreated Diabetic group. CMC: Carboxylmethyl cellulose; YB: Yoyo bitters; RB: Ruzu bitters; FB: fajik bitters; OB: Oroki bitters; FB: Fidson bitters. ALT and AST: Alanine and aspartate aminotransferases; ALP: Alkaline phosphatase

Figure 4 results show effects of different polyherbal on liver function enzymes in normal and diabetic rats. Untreated diabetic rats showed increased (*p* < 0.001) ALT by 760.9% when compared with control normal group. GLIB lowered ALT level (*p* < 0.05, 54.96%) and AST (*p* > 0.05, 30.2%) respectively when compared with control untreated diabetic group. Similarly, polyherbal YB, RB, FJB, OB and FB lowered (p < 0.05) ALT levels by 89.4, 94.1, 68.7, 82.5, and 68.7% respectively. Also, YB elevated (p > 0.05, 23.3%) AST levels in treated rats.

### The effects of polyherbal on hepatic and pancreatic lipid peroxidation levels

**Fig. 5:**
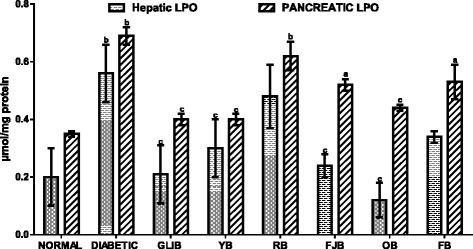
effects of polyherbals on hepatic and pancreatic lipid peroxidation (MDA) levels in normal and diabetic rats. Results represented as Mean ± S.E.M. n = 6. ^*a*^*p* < 0.05 or ^b^ *<* 0.001 different from control normal CMC group. ^*c*^*p* < 0.05 or ^d^ *<* 0.001 different from control Untreated Diabetic group. CMC: Carboxylmethyl cellulose; YB: Yoyo bitters; RB: Ruzu bitters; FB: fajik bitters; OB: Oroki bitters; FB: Fidson bitters. MDA: Malondialdehyde

Figure 5 results show effects of polyherbal on hepatic and pancreatic lipid peroxidation levels in normal and diabetic rats. In the untreated diabetic group, hepatic and pancreatic malondialdehyde (MDA) increased (*p* < 0.05) by 180 and 97% respectively. Administration of GLIB, YB, FJB, and OB lowered (p < 0.05) hepatic MDA when compared with control untreated diabetic rats. GLIB, YB, OB reduced (p < 0.05) pancreatic MDA by 42, 42, and 36.2% respectively when compared with control untreated diabetic group.

### The effects of polyherbal on hepatic and pancreatic reduced glutathione (GSH) levels

**Fig. 6:**
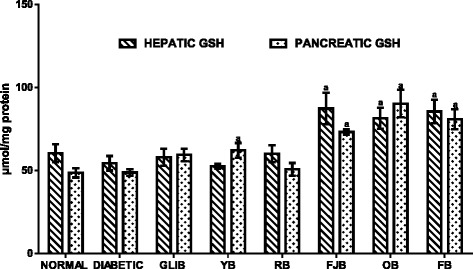
effects of polyherbals on hepatic and pancreatic reduced glutathione (GSH) levels in normal and diabetic rats. Results represented as Mean ± S.E.M. n = 6. ^*a*^*p* < 0.05 or ^b^ *<* 0.001 different from control normal CMC group. ^*c*^*p* < 0.05 or ^d^ *<* 0.001 different from control Untreated Diabetic group. CMC: Carboxylmethyl cellulose; YB: Yoyo bitters; RB: Ruzu bitters; FB: fajik bitters; OB: Oroki bitters; FB: Fidson bitters

Figure 6 results show effects of polyherbal on hepatic and pancreatic reduced glutathione (GSH) levels in normal and diabetic rats. In the untreated diabetic group, hepatic reduced glutathione (GSH) level was lowered by 9.97% when compared with control normal group. In contrast, increased (p < 0.05) hepatic and pancreatic GSH levels were obtained in rats that received FJB (60.5, 49.8%), OB (60.5, 84.9%), and FB (57.1, 65.5%) respectively when compared with control untreated diabetic group. Similarly, GLIB and YB increased although insignificantly by 21.7 and 27.2% respectively.

### Histological sections of the liver and pancreas

**Fig. 7:**
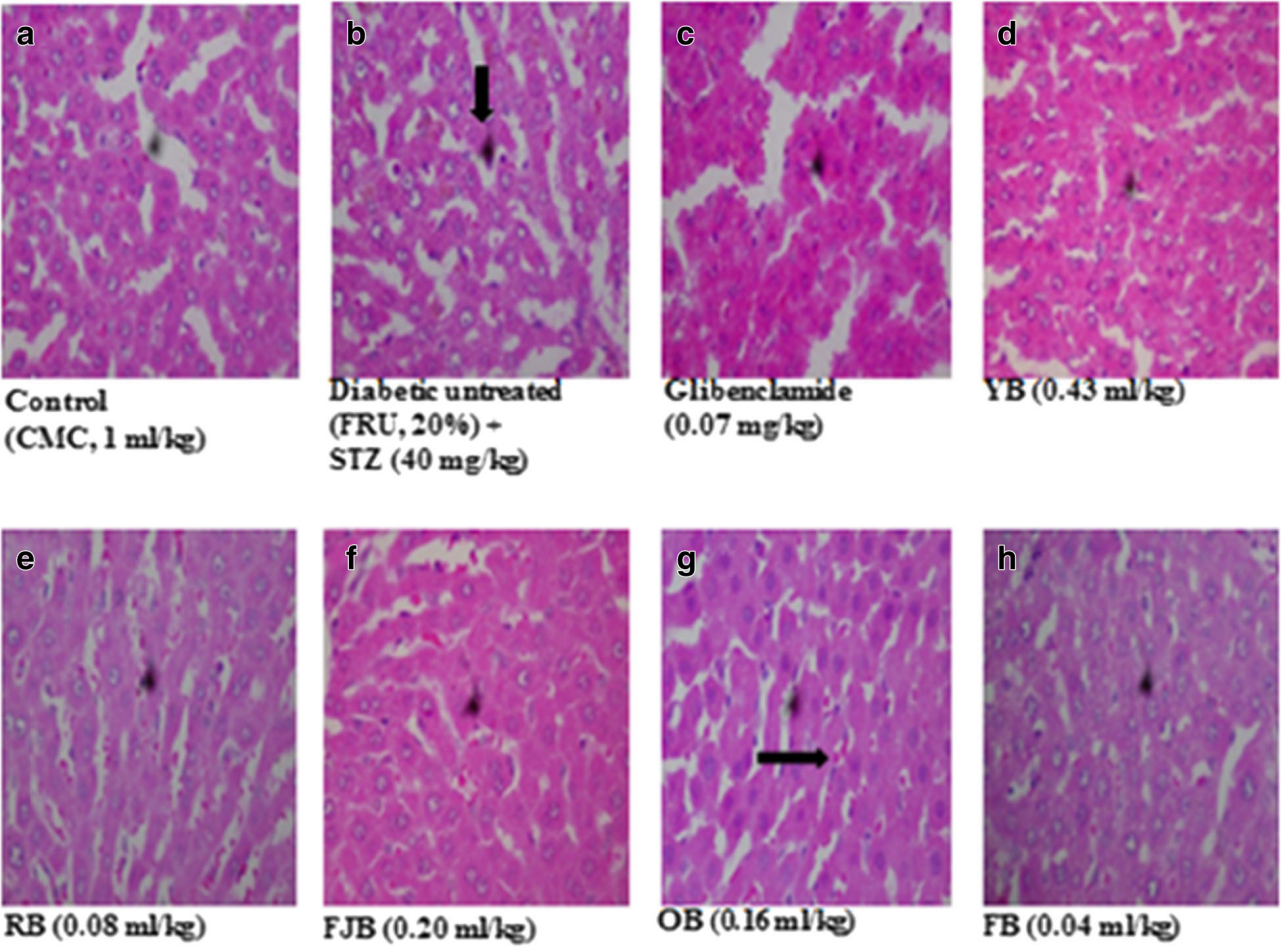
sections of LIVER show (**a**) hepatocytes arranged as radial plates. No fatty change, vascular congestion or infiltration of parenchyma by inflammatory cells is seen. (**b**) radial plates of hepatocytes. The hepatic sinusoidal congestion are packed with red cells (**c**) radial plates of hepatocytes. The hepatic sinusoidal congestion are packed with red cells. **d** hepatocytes arranged as radial plates. No fatty change, vascular congestion or infiltration of parenchyma by inflammatory cells seen. (**e**) hepatocytes arranged as radial plates. No fatty change, vascular congestion or infiltration of parenchyma by inflammatory cells seen. **f** hepatocytes arranged as radial plates. No fatty change, vascular congestion or infiltration of parenchyma by inflammatory cells is seen. **g** radial plates of hepatocytes. The hepatic sinusoidal congestion are packed with red cells. **h** hepatocytes arranged as radial plates. No fatty change, vascular congestion or infiltration of parenchyma by inflammatory cells seen. FRU: Fructose. STZ: Streptozotocin. CMC: Carboxy methyl cellulose . (H & E, mag. X 100)

**Fig. 8:**
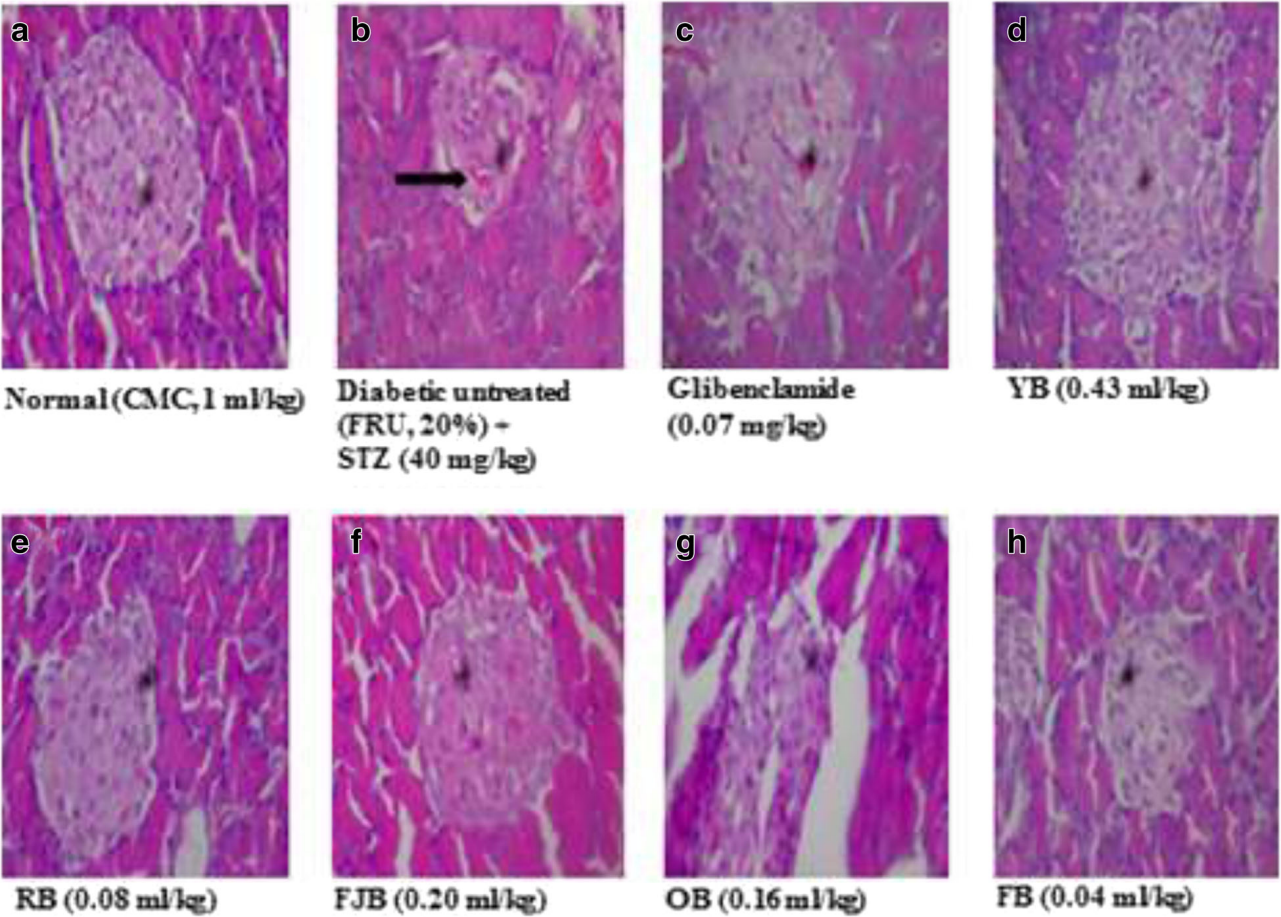
section of PANCREAS show (**a**) normocellular islets surrounded by normal appearing exocrine acini. No necrosis seen. **b** shrunk cellular islets surrounded by normal appearing exocrine acini with near-to-normal necrosis seen. **c** normocellular islets surrounded by normal appearing exocrine acini. No necrosis seen. **d** normocellular islets surrounded by normal appearing exocrine acini. **e** section of tissue shows normocellular islets surrounded by normal appearing exocrine acini. **f** section of tissue shows normocellular islets surrounded by normal appearing exocrine acini. **g** section of tissue shows normocellular islets surrounded by normal appearing exocrine acini. No necrosis seen. **h** normocellular islets surrounded by normal appearing exocrine acini. No necrosis is seen. No necrosis seen. FRU: Fructose. STZ: Streptozotocin. CMC: Carboxy methyl cellulose. (H & E, mag. X 400)

Representative photomicrographs of liver and pancreas histology of rats in all treatment groups are presented in Figs. 7 and 8 respectively. T2D rats liver and pancreas treated with HF-STZ showed sinusoidal congestion of radial plates of hepatocytes and shrunk cellular islets surrounded by normal appearing exocrine acini with near-to-normal necrosis. However, different polyherbals administered in treated rats showed some modulatory roles.

## 12iscussion

Scientists all over the world have worried over the population that roams the health centers over T2D which has become pandemic over decades [40]. Although prevalence studies present divergent reports, however suggestions are that concerted efforts are required to increase the life span of the people [41]. According to the World Health Organization, the prevalence of DM is projected to rise to 300 million within 2025 [41]. In view of the increasing prevalence, there is a growing need to develop integrated approaches toward the management and prevention of DM by exploring the potentials offered by the traditional, complementary and alternative medicines. A very large percentage of the drugs in circulation are plant derivatives [11]. The insulin injection and hypoglycemic agents continue to thrive for T2D management. However, there have been concerns over several adverse effects associated with their application. In respect, the search for effective compounds with lower side effects in treating this ailment has been on the increase [30]. Herbal medicines have in recent time contributes immensely as an alternative or complementary sources. Several studies have investigated anti-diabetic activities of various plants used in Nigeria, and have confirmed potentials for therapeutic efficacies [25, 42]. Since the manufacturers of polyherbal most especially YB, RB, FJB, OB and FB engaged in a high-quality public advertisement that has enabled high demand in pharmacies which boost their sales especially among patients with chronic diseases including diabetes and cardiovascular disorders. One major reason why this study is very important to the population is that some still indulge in drug-herb combination which may place them at a high risk since this may result in interactions which may possibly alter the bioavailability or clinical effectiveness of a conventional drug when given concurrently. Interestingly, several of the constituents of these polyherbal have scientific evidence for their uses. For instance, over thirty compounds have been synthesized from *Mangifera indica* following bioactivity guided fractionations in order to identify the active anti-diabetic constituents. Several of compounds obtained including penta-O-galloyl-β-d-glucose, 3-β taraxerol etc. have shown potentials for managing the hyperglycemic state in rodents [43, 44]. The flavonoid, epiafzelechin, was also isolated and fully characterized from the root bark of *Cassia sieberiana* and *Khaya grandifoliola* for its antioxidant activity demonstrated diabetic animals [45] and so on. Although, not all ingredients possess anti-diabetic effects, however, evidence abounds for anti-hyperglycemic effects of *Citrus aurantifolia, Aloe vera* and *Cinnamomum aromaticum* found in YB [46], *Sorghum bicolor* stem, *Khaya grandifoliola* bark, *Cassia sieberiana* root, *Alstonia cognensis* bark, *Ocimum basillicum* leaves, *Mangifera indica* leaves, *Cythula prostrate* Leaves, *Securidaca longepedunculata* root, *Saccharum officinarum* stem and water in OB [47, 48]. *Uvarie chamae, Curculigo pilosa* and *citrullis Colocythis* in RB [9, 42, 49], *Cassia alata, Citrus medica var. acida* (Roxb.)*, Aloe vera, Cassia angustifolia* in FJB [46] and g*inseng, Phyllanthus niruri, Aloe vera, Tephrosia purpurea, Eclipta alba, Swertia chirata* (Buch-Ham.)*, Casssia angustifolia, Cinnamomum zeylanicum* found in FB [50]**.** This present study investigated on the potentials of five traditional of Nigerian polyherbal remedies against high fructose-fed, streptozotocin-induced type 2 diabetes in male Wistar rats in order to ascertain their herbo-therapeutic effects. Obtaining experimental T2D in rodents has taken different routes. However, the use of high-fat diet and streptozotocin model has been on the forefront. This is because fructose-induced insulin resistance and hyperinsulinaemia in normal rats are complemented by pancreatic β-cells dysfunction that impedes insulin production. Thus, an ideal model for T2D which would closely reflect natural history and metabolic characteristics of human T2D is mimicked. Diabetic untreated rats in this study demonstrated elevated blood glucose levels (Fig. 1) accompanied by an increased lipid peroxidation in pancreas and liver respectively. In addition, the histopathology of the liver (Fig. 7) and pancreas (Fig. 8) following HF-STZ in rats resulted in sinusoidal congestion of radial plates of hepatocytes and shrunk cellular islets surrounded by normal appearing exocrine acini with near-to-normal necrosis. Similarly, there were increased serum total cholesterol, triglycerides, and low-density lipoprotein respectively in diabetic rats. These evidence of its suitability were the major reasons why HF-STZ was employed for use. Assessment of body weight at day fourth, seventh and eleventh of treatments did not show any significant change when compared with controls, although, a slight increase was observed in rats treated with OB, RB, and YB respectively. There are convergent studies that show the link between lipid and glucose profile as well as diabetic complications [51]. Polyherbal administration improved lipid parameters in diabetic rats. However, hypercholesterolemia persists in diabetic rats treated with RB and FJB respectively. In addition, RB increased LDL in treated rats. Thus, caution to avoid rebound dyslipidemia by dosage regulation and optimum compliance may enable effectiveness in RB users. In contrast, hypertriglyceremia were reduced in rats treated YB, RB, FJB, OB and FB respectively compared with controls. Treatments with YB, RB, and OB increased antioxidant lipids HDL in rats. More so, both serum TC and LDL cholesterol levels were reduced YB, FJB, OB, and FB respectively compared with control. Effects of different polyherbal on liver function enzymes in normal and diabetic rats were demonstrated. An increased alanine aminotransferase level was evident in untreated diabetic rats. Polyherbal YB, RB, FJB, OB and FB lowered ALT levels in rats. However, treatment with YB elevated AST levels in rats. Lipid peroxidation metabolite is usually measured to score damage biochemically since insulin secretion is also closely associated with lipoxygenase derived peroxides [51]. Oxidative stress has been implicated in diabetes both type 1 and 2 by increasing level of lipid peroxidation [52]. This results in generation of free radicals in pancreas and vital organs including the liver. An increase in lipid peroxidation level may initiate cellular infiltration and islet cell damage in diabetes [52]. Thus, elevation of MDA or hydroxydobenenal levels in the tissues of diabetic animals may be associated with oxidative stress. In this study, hepatic and pancreatic lipid peroxidation (LPO) levels were assessed in normal and diabetic rats and following polyherbal treatments. There were elevated hepatic and pancreatic malondialdehyde (MDA), a metabolite of LPO, in diabetic rats. Similar to anti-diabetic drug, GLIB, administration of, YB, FJB, and OB lowered reversed hepatic MDA while YB, OB reduced pancreatic MDA respectively in treated diabetic group. More so, reduced glutathione constitutes one of the antioxidant capacities that help to combat oxidative stress in diabetes by its reducing power in the cytoplasm [53]. GSH protects against toxic effects of lipid peroxidation metabolites. From the results obtained in this present study, hepatic reduced glutathione (GSH) remained low in untreated diabetic rats. In contrast, increased hepatic and pancreatic GSH levels were elevated in rats that received FJB, OB and FB respectively, although, YB shows increased but insignificantly compared with control. Further, administrations of different polyherbals however demonstrated some levels of efficacy in the treated rats, although, complete histoarchitectural status was not attained. In spite of the potential postreatment actions of these polyherbals in T2D diabetes, such subacute administration may not necessarily translate into a complete amelioration as observed in this study.

## Conclusion

The results from this present study demonstrate anti-hyperglycemic potentials of most commonly used polyherbal in Nigeria in experimental T2D rats. Although specific mechanisms of actions were not determined, however, it appears to be, in part, antioxidants mediated. Also, there is also an urgent need for caution and or monitoring because RB, FJB, and OB could elevate serum TC while RB shows a tendency to increase LDL cholesterol in rats. In addition, a holistic toxicological evaluation of these polyherbals is essential.

## Abbreviations

IR: Insulin resistance
STZ: Streptozotocin
T2D: Type 2 Diabetes
T2DM: Type 2 Diabetes Mellitus

## References

[1] Jordan SA, Cunningham DG, Marles RJ (2010). Assessment of herbal medicinal products: challenges, and opportunities to increase the knowledge base for safety assessment. Toxicol Appl Pharmacol.

[2] Keter LK, Mutiso PC (2012). Ethnobotanical studies of medicinal plants used by traditional health practitioners in the management of diabetes in lower Eastern Province, Kenya. J Ethnopharmacology.

[3] Boden G, Shulman GI (2002). Free fatty acids in obesity and type 2 diabetes: defining their role in the development of insulin resistance and β-cell dysfunction. Eur J Clin Investig.

[4] Rodriguez-Fragoso L, Reyes-Esparza J, Burchiel SW, Herrera-Ruiz D, Torres E (2008). Risks and benefits of commonly used herbal medicines in Mexico. Toxicol Appl Pharmacol.

[5] Murad MH, Coto-Yglesias F, Wang AT, Sheidaee N, Mullan RJ, Elamin MB, Montori VM (2009). Drug-induced hypoglycemia: a systematic review. J Clin Endocrin Metabolism.

[6] Li S, Zhang B, Zhang N (2011). Network target for screening synergistic drug combinations with application to traditional Chinese medicine. BMC Syst Biol.

[7] Adeyemi OS, Fambegbe M, Daniyan OR, Nwajei I (2012). Yoyo bitters, a polyherbal formulation influenced some biochemical parameters in Wistar rats. J Basic Clin Physiol Pharmacol.

[8] Oreagba IA, Oshikoya KA, Amachree M (2011). Herbal medicine use among urban residents in Lagos, Nigeria. BMC Complement Altern Med.

[9] Emordi JE, Agbaje EO, Oreagba IA, Iribhogbe OI (2016). Antidiabetic and hypolipidemic activities of hydroethanolic root extract of Uvaria chamae in streptozotocin induced diabetic albino rats. BMC Complement Altern Med.

[10] Stojanović G, Golubović T, Palić R (2009). Acinos species: chemical composition, antimicrobial and antioxidative activity. J Med Plants Res.

[11] Sharma P, Kharkwal AC, Kharkwal H, Abdin MZ, Varma A (2014). A review on pharmacological properties of Aloe vera. Int J Pharm Sci Rev Res.

[12] Radha MH, Laxmipriya NP (2015). Evaluation of biological properties and clinical effectiveness of Aloe vera: a systematic review. J traditional complementary med.

[13] Cefalu WT, Ribnicky D (2009). Modulation of insulin action by botanical therapeutics. Obesity Weight Management.

[14] Mohamed AE, Abdel-Aziz AF, El-Sherbiny EM, Mors R (2009). Anti-diabetic effect of Aloe vera juice and evaluation of thyroid function in female diabetic rats. Biosci Res.

[15] Odugbemi TO, Akinsulire OR, Aibinu IE, Fabeku PO (2007). Medicinal plants useful for malaria therapy in Okeigbo, Ondo state, Southwest Nigeria. Afr J Tradit Complement Altern Med.

[16] Benavente-Garcia O, Castillo J (2008). Update on uses and properties of citrus flavonoids: new findings in anticancer, cardiovascular, and anti-inflammatory activity. J Agric Food Chem.

[17] Calzada F, Yépez-Mulia L, Aguilar A (2006). In vitro susceptibility of Entamoeba histolytica and Giardia lamblia to plants used in Mexican traditional medicine for the treatment of gastrointestinal disorders. J Ethnopharmacol.

[18] Nabavi SF, Di Lorenzo A, Izadi M, Sobarzo-Sánchez E, Daglia M, Nabavi SM (2015). Antibacterial effects of cinnamon: from farm to food, cosmetic and pharmaceutical industries. Nutrients.

[19] Sofidiya MO, Oduwole B, Bamgbade E, Odukoya O, Adenekan S (2011). Nutritional composition and antioxidant activities of *Curculigo pilosa* (Hypoxidaceae) rhizome. Afr J Biotechnol.

[20] Murali R, Saravanan R (2012). Antidiabetic effect of d-limonene, a monoterpene in streptozotocin-induced diabetic rats. Biomedicine & Preventive Nutrition.

[21] Syamasundar KV, Singh B, Thakur RS, Husain A, Yoshinobu K, Hiroshi H (1985). Antihepatotoxic principles of Phyllanthus niruri herbs. J Ethnopharmacol.

[22] Bansal J, Kumar N, Malviya R, Sharma PK (2014). Hepatoprotective models and various natural product used in Hepatoprotective agents: a review. Pharmacognosy Communications.

[23] Balachandran P, Govindarajan R (2007). Ayurvedic drug discovery. Expert Opin Drug Discovery.

[24] Ekor M (2014). The growing use of herbal medicines: issues relating to adverse reactions and challenges in monitoring safety. Front Pharmacol.

[25] Ezuruike UF, Prieto JM (2014). The use of plants in the traditional management of diabetes in Nigeria: pharmacological and toxicological considerations. J Ethnopharmacol.

[26] Kale OE, Awodele O (2016). Safety evaluation of bon-santé cleanser® polyherbal in male Wistar rats. BMC Complement Altern Med.

[27] Kilkenny C, Browne WJ, Cuthill IC, Emerson M, Altman DG (2010). Improving bioscience research reporting: the ARRIVE guidelines for reporting animal research. PLoS Biol.

[28] Onyeaghala AA, Omotosho IO, Shivashankara AR (2015). Cytotoxicity of various fractions of compounds extracted from Yoyo bitters on human cervical Cancer cells. European J Med Plants.

[29] Proestos C, Sereli D, Komaitis M (2006). Determination of phenolic compounds in aromatic plants by RP-HPLC and GC-MS. Food Chem.

[30] Okolie AC, Kale OE, Osilesi O. Chemoprotective effects of butanol fraction of Buchholzia Coriacea (Capparidaceae) against type 2 diabetes and oxidative stress in male Wistar rats. Biosci Rep. BSR20170665. 2017; 10.1042/BSR20170665. 10.1042/BSR20170665 PMC6379225 28790167

[31] Wilson R, Islam M (2012). Fructose-fed Streptozotocin injected rat; an alternative model for type 2 diabetes.

[32] Reitman S, Frankel SA (1957). Colorimetric method for the determination of serum glutamate oxaloacetate and pyruvate transaminases. Am J Clin Pathol.

[33] Roy AV (1970). Rapid method for determining alkaline phosphatase activity in serum with thymolphthalein monophosphate. Clin Chem.

[34] Trinder P (1969). Quantitative determination of triglyceride using GPO-PAP method. Annals Clin Biochem.

[35] Fossati P, Prencipe L, Berti G (1983). Enzymic creatinine assay: a new colorimetric method based on hydrogen peroxide measurement. Clin Chem.

[36] Warnick GR, Albers JJ (1978). A comprehensive evaluation of the heparinmanganese precipitation procedure for estimating high density lipoprotein cholesterol. J Lipid Res.

[37] Friedewald WT, Levy RI, Fredrickson DS (1972). Estimation of the concentration of low-density lipoprotein cholesterol in plasma, without use of the preparative ultracentrifuge. Clin Chem.

[38] Beutler E, Duron O, Kelly BM (1963). Improved method for the determination of blood glutathione. J Lab Clin Med.

[39] Varshney R, Kale RK (1990). Effect of calmodulin antagonist on radiation induced lipid peroxidation in microsomes. Inter J Radiation Biol.

[40] Shaw JE, Sicree RA, Zimmet PZ (2010). Global estimates of the prevalence of diabetes for 2010 and 2030. Diabetes Res Clin Pract.

[41] Whiting DR, Guariguata L, Weil C, Shaw J (2011). IDF diabetes atlas: global estimates of the prevalence of diabetes for 2011 and 2030. Diabetes Res Clin Pract.

[42] Gbolade AA (2009). Inventory of antidiabetic plants in selected districts of Lagos state Nigeria. J Ethnopharmacology.

[43] Mohan CG, Viswanatha GL, Savinay G, Rajendra CE, Halemani PD (2013). 1, 2, 3, 4, 6 Penta-O-galloyl-β-d-glucose, a bioactivity guided isolated compound from Mangifera indica inhibits 11β-HSD-1 and ameliorates high fat diet-induced diabetes in C57BL/6 mice. Phytomedicine.

[44] Sangeetha KN, Sujatha S, Muthusamy VS, Anand S, Nithya N, Velmurugan D, Lakshmi BS (2010). 3β-taraxerol of Mangifera indica, a PI3K dependent dual activator of glucose transport and glycogen synthesis in 3T3-L1 adipocytes. Biochimica et Biophysica Acta (BBA)-General Subjects.

[45] Zhu F (2016). Chemical composition and health effects of Tartary buckwheat. Food Chem.

[46] Sreekeesoon DP, Mahomoodally MF (2014). Ethnopharmacological analysis of medicinal plants and animals used in the treatment and management of pain in Mauritius. J Ethnopharmacol.

[47] Farrar JL, Hartle DK, Hargrove JL, Greenspan P (2008). A novel nutraceutical property of select sorghum (Sorghum bicolor) brans: inhibition of protein glycation. Phytother Res.

[48] Patel DK, Kumar R, Laloo D, Hemalatha S (2012). Natural medicines from plant source used for therapy of diabetes mellitus: an overview of its pharmacological aspects. Asian Pacific J Tropical Disease.

[49] Soladoye MO, Chukwuma EC, Owa FP (2012). An ‘Avalanche’of plant species for the traditional cure of diabetes mellitus in south-western Nigeria. J Nat Prod Plant Resour.

[50] Boaduo NKK, Katerere D, Eloff JN, Naidoo V (2014). Evaluation of six plant species used traditionally in the treatment and control of diabetes mellitus in South Africa using in vitro methods. Pharm Biol.

[51] Tuhin RH, Begum MM, Rahman MS, Karim R, Begum T, Ahmed SU, Begum R, Mostofa R, Hossain A, Abdel-Daim M, Begum R (2017). Wound healing effect of Euphorbia hirta Linn.(Euphorbiaceae) in alloxan induced diabetic rats. BMC complementary and alternative medicine.

[52] Brownlee M (2001). Biochemistry and molecular cell biology of diabetic complications. Nature.

[53] Rahimi R, Nikfar S, Larijani B, Abdollahi M (2005). A review on the role of antioxidants in the management of diabetes and its complications. Biomed Pharmacother.

